# Sex-Dependent Dyslipidemia and Neuro-Humoral Alterations Leading to Further Cardiovascular Risk in Juvenile Obesity

**DOI:** 10.3389/fnut.2020.613301

**Published:** 2021-02-12

**Authors:** Estefania Simoes, Joanna Correia-Lima, Elie Leal de Barros Calfat, Thais Zélia dos Santos Otani, Daniel Augusto Correa Vasques, Victor Henrique Oyamada Otani, Pamela Bertolazzi, Cristiane Kochi, Marilia Seelaender, Ricardo Riyoiti Uchida

**Affiliations:** ^1^Cancer Metabolism Research Group, University of São Paulo, São Paulo, Brazil; ^2^Mental Health Department, Santa Casa de São Paulo School of Medical Sciences, São Paulo, Brazil; ^3^Physiology Department, Santa Casa de São Paulo School of Medical Sciences, São Paulo, Brazil; ^4^Faculdade de Medicina, University of São Paulo, São Paulo, Brazil; ^5^Laboratórios de Inventigação Médica 26, Hospital das Clínicas of the University of São Paulo, São Paulo, Brazil

**Keywords:** obesity, metabolic syndrome, dyslipidemia, hormones, neuropeptides

## Abstract

**Objective:** Childhood obesity is a growing concern as the World Health Organization (WHO) states that ~10% of adolescents worldwide are overweight or obese. This condition is the reflex of energy imbalance between the calories consumed and those expended. Sex-related responses associated with dyslipidemia, hormonal alterations, and neuro-humoral disruptions in childhood obesity are the focus of the present investigation.

**Methods:** Ninety-two Brazilian adolescents were enrolled and divided between obese and eutrophic groups. Obesity was assessed using body mass index Z-score according to age and weight. Anthropometrical analyses, blood pressure, blood lipids, metabolism-regulating hormones, and neuropeptides were carried out.

**Results:** Systolic blood pressure was higher in female and male patients with obesity. Obese females presented alterations in lipid profile and an augment of cardiovascular disease prediction ratios TC/HDL, TG/HDL, LDL/HDL, and VLDL/HDL. The levels of leptin, GIP, and neuropeptide showed sex-dimorphism in obesity. The obese adolescents presented increased levels of circulating insulin, c-peptide, amylin, glucagon, and GLP-1. Correlation analysis showed significant linearity between body mass index, blood pressure, lipids, lipoproteins, hormones, and neuropeptides content.

**Conclusions:** Our data support an existing link associating hypertension, dyslipidemia, and neuro-hormonal imbalance in childhood obesity. We also described a sex-dependent pattern in childhood obesity-associated dyslipidemia and blood pressure in female patients with obesity solely.

## Introduction

Obesity is a consequence of energy imbalance between the calories consumed and those expended and is associated with several health problems. Obesity prevalence worldwide has drastically increased over the last years, augmenting the risk of mortality due to comorbidities (1, 2). Childhood obesity is a growing concern: in 2019, the World Health Organization (WHO) estimated that ~10% of adolescents worldwide are overweight or obese (3). Excessive adiposity in the young population conduces into adult obesity, imposing markedly increased risk for metabolic disturbance and cardiovascular disease, as a consequence of hypertension, lipid disturbances, or hormonal impaired status in adult life (4–6). Abnormalities in serum lipid levels (dyslipidemia) and hypertension are inextricably linked risk factors for cardiovascular diseases (7–9). Although several studies with adults reported that these factors act in an age- and sex-dependent manner, sexual dimorphism in the juvenile population is still poorly studied (10). The pathophysiology of obesity-related hypertension is associated with overactivity of the sympathetic nervous system (SNS), and sodium retention, among other alterations (11). The gut-brain axis, a complex system driver of body weight homeostasis, has recently emerged as a major culprit for obesity-associated metabolic syndrome. Peripheral appetite hormones (insulin, leptin, glucagon-like peptide 1 (GLP-1), among others) are sensed by the hypothalamus- and do not only participate in appetite regulation, but also influence SNS activation (12). In adults, alterations in hormonal levels due to obesity, such as hyperinsulinemia or hyperleptinemia seem to be responsible for SNS overactivity and to play an important role in blood pressure regulation (13, 14). Hyperinsulinemia caused by insulin resistance stimulates Sodium reabsorption, enhances Sodium retention, leading to hypertension (15). Leptin contributes to an increased plasma volume by acting on neurons expressing either neuropeptide Y (NPY) or alpha-melanocyte stimulating hormone (α-MSH) (16).

Therefore, the aim of the present study was to elucidate the possible link comprising hypertension, dyslipidemia, hormonal alterations, and neuro-humoral disruptions. To our knowledge, this is the first study to simultaneously address anthropometrical measurements, lipid and lipoprotein profile, hormone and neuropeptide concentration, with a special focus on sex-dependent responses in a population with obesity and eutrophic juvenile cohort.

## Materials and Methods

### Participants

Ninety-two adolescents were recruited between April 2014 and July 2016 at the Children Obesity Outpatient Clinic of the Santa Casa de Misericordia Hospital in São Paulo (SCMHSP) in São Paulo, Brazil. We included adolescents between ages 11 and 18 years, constituting a control group with a BMI z-score above or equal to one and an overweight group with a BMI z-score greater or equal to two. BMI z-scores calculated per the 2006 WHO (World Health Organization) growth standard, which will be called BMI Z-score (17). The adolescents were divided into Eutrophic [Female (EUF): *n* = 24, BMI z-score = −0.38 ± 0.18; Male (EUM): *n* = 25, BMI *z*-score = −0.27 ± 0.19] and Obesity [Female (OBF): *n* = 22, BMI *z*-score = 2.69 ± 0.11; Male (OBM): *n* = 21, BMI *z*-score = 2.83 ± 0.12] groups. The study was approved by the SCMHSP Ethics Committee (CAAE: 24552413.2.0000.5479). All participants and their parents signed an informed consent prior to engaging in the study, according to the Declaration of Helsinki. The exclusion criteria were: current or previous clinical, neurological, and psychiatric illness, as well as history of cranioencephalic trauma, seizure or previous neurosurgery. Anthropometrical data of the entire cohort is summarized in Table 1.

**Table 1:** Anthropometrical data of eutrophic and obesity cohort.

	**Eutrophic**		**Obesity**		**Two-way ANOVA**		
	**Female**	**Male**	**Female**	**Male**	**Sex**	**Obes**.	**SxO**
Age (years)	14.46 ± 0.43	14.20 ± 0.39	13.82 ± 0.43	13.67 ± 0.43	0.6271	0.1663	0.8992
Weight (Kg)	47.25 ± 1.96	51.23 ± 2.49	83.65 ± 3.29	86.76 ± 3.98	0.2345	**<0.0001**	0.8845
Height (m)	1.58 ± 0.02	1.64 ± 0.03	1.62 ± 0.01	1.66 ± 0.02	0.5104	0.1674	0.7003
BMI (kg/m^2^)	18.80 ± 0.49	18.84 ± 0.48	31.99 ± 1.10	31.32 ± 0.89	0.6793	**<0.0001**	0.6499
BMI *Z*-score	−0.38 ± 0.18	−0.27 ± 0.19	2.69 ± 0.11	2.83 ± 0.12	0.4429	**<0.0001**	0.9540
YFAS	1.63 ± 0.39	1.63 ± 0.36	1.50 ± 0.24	2.43 ± 0.39	0.1937	0.3412	0.1937
BES	8.83 ± 1.54	4.96 ± 0.81	9.68 ± 1.55	11.33 ± 1.36	0.4091	**0.0085**	0.0422
SBP (mmHg)	106 ± 1.62	110.8 ± 1.85	119.2 ± 3.51	123.2 ± 3.59	0.1018	**<0.0001**	0.8734
DBP (mmHg)	75.26 ± 2.15	72.96 ± 2.58	77.22 ± 2.26	78.95 ± 2.28	0.9036	0.0984	0.3994

*Eutrophic female, n = 24; Eutrophic male, n = 25; Obesity female, n = 22; Obesity male, n = 21. BMI, Body Mass Index; YFAS, Yale Food Addiction scale; BES, Binge Eating Scale score; SBP, systolic blood pressure; DBP, diastolic blood pressure; Obes., Obesity factor; SxO, Interaction between factors (sex and obesity). Data presented as mean ± sem. Significance was detected using two-way ANOVA. Significant p-values appear in bold*.

### Blood Collection and Clinical Measurements

Approximately 6 mL of blood was collected by a trained health professional from the SCMHSP after overnight fasting (BD Vacutainer® Tubes). Blood pressure measurements were taken in the upper arm by a mercury sphygmomanometer, considering Korotkoff sounds disappearance criteria. Age at menarche was recorded for the participating female adolescents.

### Blood Lipid and Lipoprotein Levels

Santa Casa de Misericordia Hospital performed the measurement of fasting serum concentration of total cholesterol (TC), high-density lipoprotein cholesterol (HDL), low-density lipoprotein cholesterol (LDL), Very-low-density lipoprotein cholesterol (VLDL), and triglycerides (TG) (Cobas® C501, Diagnostic Roche).

### Questionnaire Assessments

The Yale Food Addiction Scale (YFAS) was assessed considering a 25-item questionnaire designed to identify the eating patterns that exhibit signs of addiction to high-fat and/or high-sugar foods (18). The Binge Eating Scale (BES) was assessed using a 16-item questionnaire to identify the presence of binge eating behavior indicative of an eating disorder. The questions are based on both behavioral characteristics (e.g., amount of food consumed) and the emotional, cognitive response, (e.g., guilt or shame) (19).

### Circulating Protein Analysis

Serum Samples from the experimental groups were analyzed by the Luminex®xMAP™ technology or Enzyme-Linked Immunosorbent Assay (ELISA). Multiplex assays were performed according to the manufacture's protocol. Luminex®xMAP™ technology commercial Merck Millipore kits references: Human Metabolic Hormone Magnetic Bead Panel (Cat. #HMHEMAG-34K) and Human Neuropeptide Magnetic Bead Panel (Cat. #HNPMAG-35K). Serum Samples were incubated with the mixture of Multiplex Magplex microspheres and covered with the specific antibodies. The detection of target antigens bound to the microspheres was performed with a mixture of biotinylated capture antibodies incubation and followed by incubation with streptavidin labeled with phycoerithrin. The microspheres were analyzed with the phycoerithrin Magpix® instrument (Life Technologies, Grand Island, NY, USA). Protein quantification was preceded by the use of the equipment software (xPONENT® 4.2) and the obtained data were analyzed in the MILLIPLEX® Analyst 5.1 Software.

Neuropeptide Y (NPY) and Melanin Concentrating Hormone (MCH) were quantified with the Enzyme-Linked Immunosorbent Assay (ELISA) following the manufacturer's protocol (Elisa Millipore Cat. #EZHNPY-25K, Merck Millipore and Enzyme Immunoassay Phoenix Pharmaceuticals Cat. #EK-070-47, respectively). Serum Samples were incubated on the immunoplate, coated by a pre-tittered amount of anchor antibodies, and the non-specific binding sites blocked. The detection of target antigens bound to the microspheres was performed with a mixture of biotinylated capture antibodies incubation and followed by incubation with streptavidin labeled with horseradish peroxidase (SA-HRP). The enzyme activity was measured spectrophotometrically by the increased absorbency at 450 nm. The analyte concentration was determined interpolating test sample response data from a reference curve.

### Statistical Analysis

Data are expressed as mean ± SEM (standard error of the mean). For this population (150 patients), the ideal sample size is 85 patients in a 95% of confidence level (margin of error of 7%). Gaussian distribution test was employed for all continuous variables (Kolmogorov–Smirnov Test). Non-parametric data were transformed into normal distribution by applying a mathematical function (log or square root) to make the statistical analyses feasible with the Two-way ANOVA test with multiple comparisons possible. Differences were detected using the Two-way ANOVA test with multiple comparisons and *post hoc* pairwise comparisons Tukey's test, when appropriate. Spearman's or Pearson's correlation coefficient and linear regression were used to assess the simple relationship between the variables. Significance level was set at *p* < 0.05. All statistical tests were performed using the GraphPad Prism 7.0.

## Results

**Figure 1:**
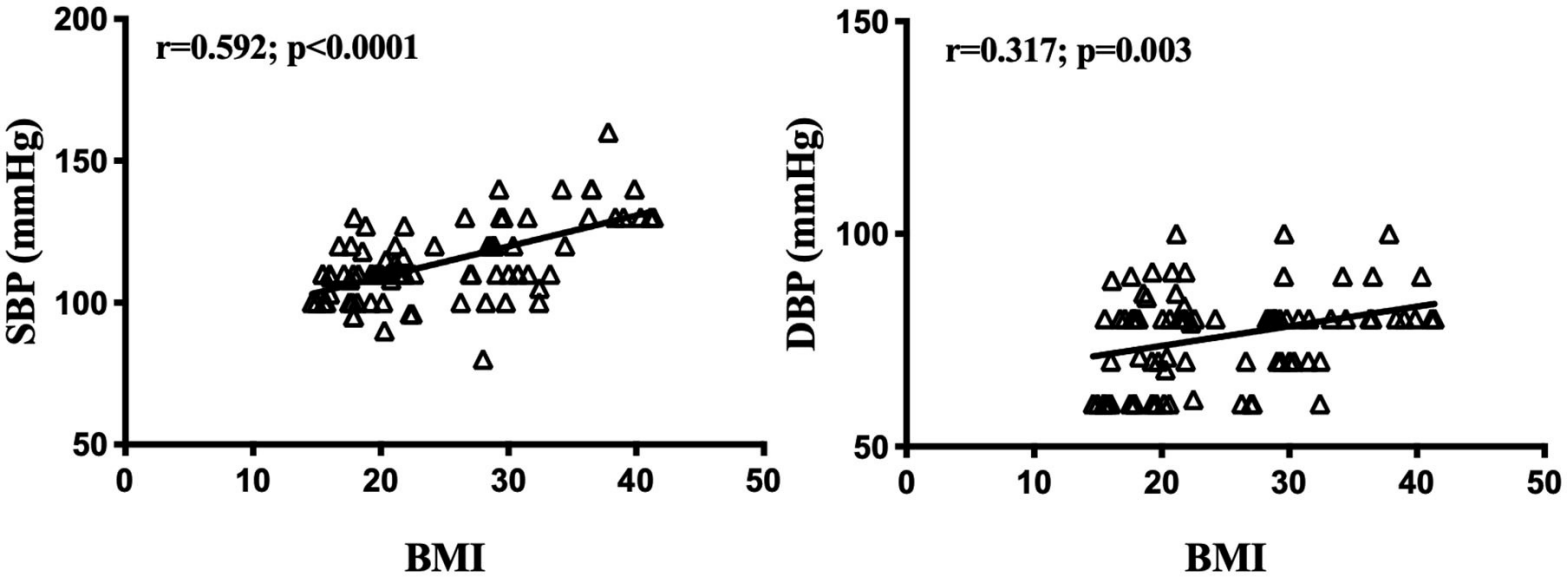
Blood pressure and body mass index z-score correlations. BMI z-score, Body mass index z-score; SBP, systolic blood pressure; DBP, diastolic blood pressure. Significance correlation between factors was tested using the Spearman or Pearson correlation test. Significant correlations in bold (*p* < 0.05).

Anthropometrical characteristics of the entire cohort (*n* = 92) are illustrated in Table 1. There were no differences in distributions of age or height (*p* > 0.05). Weight, Body mass index (BMI), and BMI *z*-score of the obesity group were higher compared to those of the eutrophic group, for both sexes (*p* < 0.0001). Average menarche age was 11.63 ± 0.26 years among eutrophic girls, and 11.82 ± 0.33 in obese girls (*p* = 0.644). The Binge Eating Scale (BES, *p* = 0.0085) showed higher scores in both sexes in adolescents with obesity, indicating the presence of eating disorder. However, the Yale Food Addiction Scale (YFAS, *p* = 0.3412) presented no difference among groups. Blood pressure (BP) measurement showed systolic blood pressure (SBP) to be obesity-dependent (*p* < 0.0001), whereas *post-hoc* Tukey's test revealed significantly higher levels in obese females (119.2 ± 3.51 mmHg, *p* = 0.0039) and males (123.2 ± 3.59 mmHg, *p* = 0.0071), compared with their eutrophic counterparts (106 ± 1.62 mmHg and 110.8 ± 1.85 mmHg, respectively). No statistical differences were found among groups for diastolic blood pressure (DBP). Nevertheless, SBP (*r* = 0.487; *p* < 0.001) and DBP (*r* = 0.289; *p* = 0.008) were positively correlated with BMI Z-score in the entire cohort (Figure 1).

### Childhood Obesity Leading to a Dyslipidemic Profile

**Figure 2:**
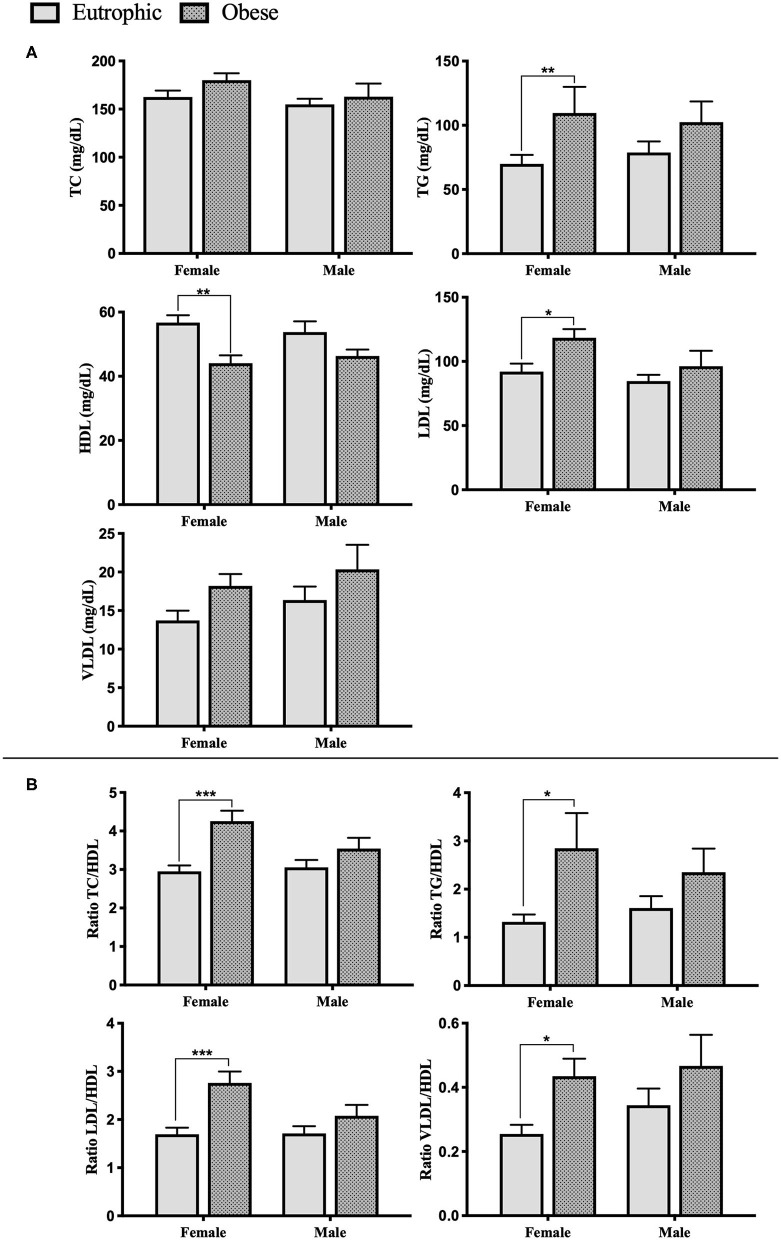
Serum lipid profile of enrolled patients. **(A)** Serum lipid and lipoproteins levels. **(B)** Cardiovascular diseases prediction ratios. Eutrophic female, *n* = 24; Eutrophic male, *n* = 25; Obesity female, *n* = 22; Obesity male, *n* = 21. TC, Total Cholesterol; TG, Triglycerides; HDL, high-density lipoprotein cholesterol; LDL, low-density lipoprotein cholesterol; VLDL, very-low-density lipoprotein cholesterol. Data presented as mean ± sem. Significance between the groups was tested using the Tukey's post–test; *p < 0.05; **p < 0.005; ***p < 0.001.

Circulating lipid levels in childhood obesity group and the eutrophic counterparts are summarized in Figure 2 (See mean levels in Supplementary Table 1). ANOVA two-way analysis showed that total Cholesterol (TC) content was not significantly different among groups (*p* > 0.005), while triglycerides (TG) were increased in the population with obesity compared to eutrophic groups (*p* = 0.0064). Obesity was found to be associated with lower levels of high-density lipoprotein cholesterol (HDL) (*p* = 0.008), and increased low-density lipoprotein cholesterol (LDL) (*p* = 0.038). *Post-hoc* multiple comparison test revealed that this difference solely appears when comparing obese girls and eutrophic girls (Figure 2A). No significant differences were found in regard to very-low-density lipoprotein cholesterol (VLDL) content among groups. The ratios between lipids and lipoproteins, TC/HDL (*p* = 0.0003), TG/HDL (*p* = 0.0087), LDL/HDL (*p* = 0.0004), and VLDL/HDL (*p* = 0.014) were increased in obese girls, compared to eutrophic girls, what indicates higher risk of developing cardiovascular diseases, such as hypertension (Figure 2B). These data demonstrate that dyslipidemia in childhood obesity is more prominent in female adolescents.

**Figure 3:**
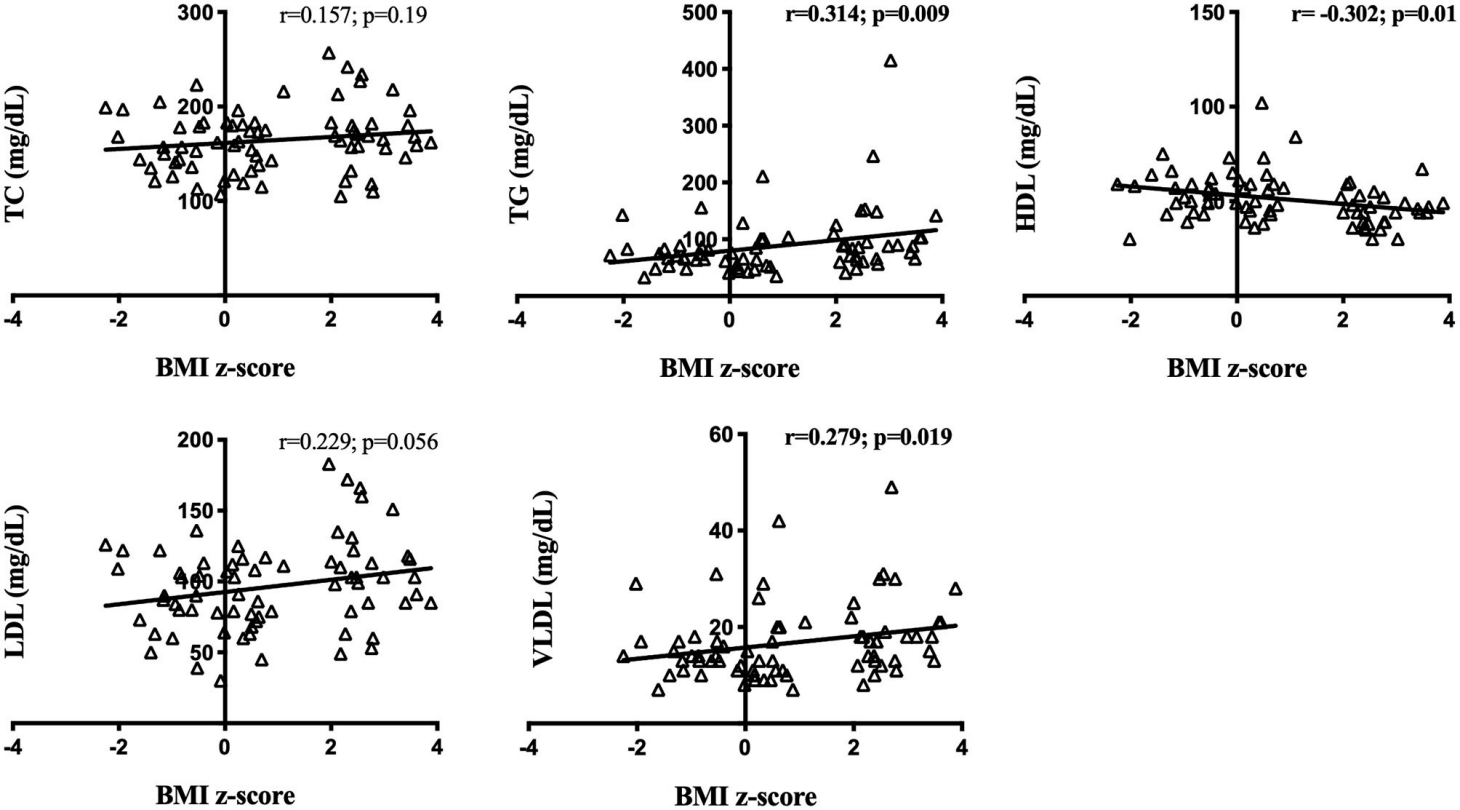
Circulating lipid content and body mass index z-score correlations in the entire cohort. BMI *z*-score: Body mass index *z-*score. TC, Total Cholesterol; TG, Triglycerides; HDL, high-density lipoprotein cholesterol; LDL, low-density lipoprotein cholesterol; VLDL, very-low-density lipoprotein cholesterol. Significance correlation between factors was tested using the Spearman or Pearson correlation test. Significant correlations in bold (*p* < 0.05).

Correlation analysis with BMI z-score of the entire cohort showed that HDL was negatively correlated with BMI Z-score (*r* = −0.302, *p* = 0.01), while TG (*r* = 0.314, *p* = 0.009) and VLDL (*r* = 0.279, *p* = 0.019) showed a positive linear correlation with BMI *z*-score (Figure 3). Moreover, correlation analysis of lipids, lipoproteins, and blood pressure demonstrated linearity only for SBP and TG (*r* = 0.282, *p* = 0.023), considering all the enrolled patients (Supplementary Table 2).

### Circulating Hormones Aggravating Hypertension and Dyslipidemia

The ANOVA two-ways analysis of anorexigenic and orexigenic circulating hormone content are shown in Table 2. Sex-dimorphism in hormone concentration was observed between eutrophic females (EUF) and males (EUM), and between obese female (OBF) and male (OBM) patients. Gastric inhibitory polypeptide (GIP) was significantly increased in EUM compared to EUF (*p* = 0.012). Leptin was higher in EUF relative to EUM (*p* < 0.0001), as well as higher in OBF, compared to OBM (*p* < 0.0001).

**Table 2:** Serum hormone content in the entire cohort.

	**Eutrophic**		**Obesity**		**Two-way ANOVA**		
**pg/ml**	**Female**	**Male**	**Female**	**Male**	**Sex**	**Obes**.	**SxO**
Insulin	1,678 ± 67.54	1,659 ± 46.86	2,772 ± 131	2,704 ± 133.8	0.659	**<0.0001**	0.806
Leptin	9,610 ± 1021	4,519 ± 308.1	34,623 ± 4,638	20,904 ± 2,834	**<0.0001**	**<0.0001**	0.416
Amylin	154.1 ± 10.71	179.6 ± 13.99	218.2 ± 16.94	230.4 ± 14.54	0.186	**0.0001**	0.636
C-peptide	438.7 ± 49.88	502.7 ± 44.57	1,224 ± 113.3	1,049 ± 112.7	0.505	**<0.0001**	0.153
Ghrelin	60.87 ± 10.84	53.34 ± 11.05	77 ± 12	69.84 ± 10.06	0.494	**0.0407**	0.623
GIP	49.23 ± 3.58	66.61 ± 7.85	54.45 ± 3.27	61.2 ± 3.89	**0.012**	0.457	0.358
GLP-1	76.05 ± 3.04	76.05 ± 2.69	94.08 ± 4.57	103.2 ± 2.73	0.145	**<0.0001**	0.175
Glucagon	142.6 ± 3.39	145.3 ± 2.36	155.8 ± 6.16	157.1 ± 4.89	0.474	**0.0022**	0.839
PP	69.28 ± 5.22	75.64 ± 6.61	79.18 ± 9.40	81.16 ± 5.49	0.227	0.166	0.991
PYY	325.2 ± 6.49	324.7 ± 6.98	329.7 ± 5.42	332.7 ± 5.45	0.854	0.252	0.756

*Eutrophic female, n = 24; Eutrophic male, n = 25; Obesity female, n = 22; Obesity male, n = 21. Obes., Obesity factor; SxO, Interaction between factors (sex and obesity); GIP, gastric inhibitory polypeptide; GLP-1, glucagon-like peptide-1; PP, pancreatic polypeptide; PYY, peptide tyrosine tyrosine. Data are presented as mean ± sem. Significance between groups was tested using two-way ANOVA test. Significant difference p-values appear in bold*.

Hormone serum content in female and male adolescents with obesity was different relative to the respective eutrophic counterparts. Girls with obesity presented a hormonal profile characterized by increased levels of circulating insulin (*p* < 0.0001), leptin (*p* < 0.0001), c-peptide (*p* < 0.0001), amylin (*p* = 0.0018), glucagon (*p* = 0.0195), and glucagon-like peptide-1 (GLP-1, *p* = 0.0001), relative to eutrophic controls. Similarly, in boys with obesity, there was a higher expression of insulin (*p* <0.0001), leptin (*p* <0.0001), c-peptide (*p* < 0.0001), amylin (*p* = 0.0013), glucagon (*p* = 0.04), GLP-1 (*p* < 0.0001), and ghrelin (*p* = 0.07), compared to the eutrophic group. No differences were found for pancreatic polypeptide (PP), nor for peptide tyrosine tyrosine (PYY) content, neither in relation to sex nor obesity (*p* > 0.005). Furthermore, Pearson and Spearman analyses revealed inner correlations between hormones, blood pressure, and circulating lipid content (see Supplementary Table 3). SBP was positively correlated to insulin (*r* = 0.422; *p* <0.0001), leptin (*r* = 0.340; *p* = 0.0015), C-peptide (*r* = 0.494; *p* <0.0001), amylin (*r* = 0.398; *p* = 0.0002), GLP-1 (*r* = 0.415; *p* <0.0001), and GIP (*r* = 0.223; *p* = 0.040). DBP positively correlated with insulin (*r* = 0.22; *p* = 0.044) and C-peptide (*r* = 0.225; *p* = 0.039). TC and LDL were found to correlate with glucagon (*r* = 0.246; *p* = 0.037 and *r* = 0.269; *p* = 0.023, respectively). Nevertheless, TG showed a positive correlation to insulin (*r* = 0.459; *p* = 0.0001), leptin (*r* = 0.254; *p* = 0.035), c-peptide (*r* = 0.451; *p* = 0.0001), and GLP-1(*r* = 0.293; *p* = 0.014). HDL was negatively correlated to insulin (r = −0.396; p = 0.0008), leptin (r = −0.239; p = 0.043), c-peptide (r = −0.397; p = 0.0005), and GLP-1(*r* = −0.239; *p* = 0.043). Finally, VLDL was found to correlate with insulin (*r* = 0.322; *p* = 0.008), C-peptide (*r* = 0.253; *p* = 0.034), and GLP-1(*r* = 0.258; *p* = 0.029).

### Circulating Neuropeptides Linked to Neuro-Humoral Alterations

ANOVA (see Table 3) revealed that neuropeptide content is markedly altered in obesity, with changes in the expression of α-MSH (α-Melanocyte-stimulating hormone, *p* = 0.0397), neurotensin (*p* = 0.0018), and melanin-concentrating hormone (MCH, *p* = 0.0003). Neuropeptide Y (NPY) levels were affected by the interaction of both factors, sex and obesity (*p* = 0.0411).

**Table 3:** Entire cohort neuropeptides analysis in serum samples.

	**Eutrophic**		**Obesity**		**Two-way ANOVA**		
**pg/ml**	**Female**	**Male**	**Female**	**Male**	**Sex**	**Obes**.	**SxO**
α-MSH	11,188 ± 2,115	10,010 ± 2,521	8,513 ± 1,821	6,958 ± 1,781	0.333	**0.0397**	0.810
β- Endorphin	11,606 ± 3,670	11,625 ± 4,061	8,995 ± 2,161	10,339 ± 2,802	0.923	0.266	0.691
Neurotensin	482.9 ± 54.16	483.3 ± 72.89	318.5 ± 29.48	358.9 ± 30.64	0.545	**0.0018**	0.298
Orexin A	10,574 ± 2072	11,576 ± 2,901	7,033 ± 882.4	8,358 ± 1,804	0.912	0.075	0.648
Oxytocin	4,083 ± 991.9	5,502 ± 1,574	2,521 ± 441.8	3,215 ± 767.5	0.697	0.052	0.805
Subt. P	161.3 ± 17.76	134.1 ± 13.91	117.7 ± 13.21	136.2 ± 17.50	0.819	0.102	0.164
NPY	43.21 ± 4.06	39.05 ± 2.68	38.61 ± 2.85	47.64 ± 2.74	0.446	0.531	**0.0411**
MCH (ng/ml)	2.67 ± 0.39	2.99 ± 0.26	2.18 ± 0.27	1.86 ± 0.08	0.539	**0.0003**	0.105

*Eutrophic female, n = 11–24; Eutrophic male, n = 16–25; Obesity female, n = 19–22; Obesity male, n = 14–21. Obes., Obesity factor; SxO, Interaction between factors (sex and obesity); α-MSH, α-Melanocyte-stimulating hormone; NPY, neuropeptide Y; MCH, melanin-concentrating hormone. Data presented as mean ± sem. Significance between groups was tested using two-way ANOVA test. Significant difference p-values in bold*.

Comparing the eutrophic female and male patients *post hoc* analyses, we failed to find significant differences in neuropeptide expression (*p* > 0.05). Nevertheless, when we compared obese male and female adolescents we observed a different pattern of the alterations in circulating neuropeptide markers. Obesity in girls leads to decreased levels of neurotensin relative to eutrophic girls (*p* = 0.0035), while boys with obesity showed lower levels of MCH (*p* = 0.0002) and higher NPY content (*p* = 0.06), as compared to eutrophic boys. Moreover, NPY expression presented sex-dimorphism, boys with obesity showing higher levels than girls with obesity (*p* = 0.05). Supplementary Table 4 shows the correlation analysis for neuropeptides, blood pressure, and lipoprotein. Neurotensin was negatively correlated to SBP (*r* = −0.249; *p* = 0.039) and TC (*r* = −0.296; *p* = 0.024), MCH was linearly negative to DBP (*r* = −0.299; *p* = 0.005) and NPY to LDL (*r* = −0.256; *p* = 0.032).

## Discussion

In order to elucidate possible coexisting and synergizing mechanisms driving obesity-associated comorbidities, the present study evaluated cohort of obese Brazilian adolescents and the eutrophic counterparts. We assessed simultaneously anthropometrical measurements (blood pressure), the circulating lipid and lipoprotein profile, and the hormonal status and serum neuropeptide content. Considering that childhood obesity is strongly associated with a higher risk to develop early cardiovascular and metabolic diseases, these new insights and the correlation results may detect existing links between well-known obesity related risk factors.

In terms of anthropometrical measurements, our data showed that the young population with obesity had a higher weight and body mass index relative to their eutrophic counterparts, as expected. In regard to blood pressure (BP) measurement, even if all the groups presented normotensive values, both obese female and male adolescents showed significantly increased systolic blood pressure. Moreover, correlation analysis revealed a positive linearity between BMI *z*-score and SBP, as well as between BMI *z*-score and DBP. Although BMI *z*-score is not a direct marker of adiposity or fat distribution, these results indicate that an increased BMI *z*-score in adolescents with obesity could be a risk factor to develop early hypertension (4, 20). Several studies showed that an increased blood pressure in obesity results from increased circulating plasma volume and cardiac output. Through many mechanisms, including sympathetic nervous system (SNS) overactivity, Sodium retention, salt sensitivity, dyslipidemia, and insulin resistance (21–23). As lipid abnormalities were found to predict hypertension (11, 24), we analyzed circulating lipids and lipoproteins in our obesity and eutrophic juvenile populations. Our data show that dyslipidemia is clearly committing obese adolescents, especially girls, who presented increased levels of TG and LDL, whereas HDL was lower in comparison to the eutrophic females. It is well-known that the incidence of obesity is associated with hypertriglyceridemia or reduced HDL levels, nonetheless, sexual dimorphism in juvenile obesity has been poorly investigated (25–27). Aiming at studying an inner link between BMI *z*-score, BP, lipid, and lipoproteins, we carried out correlation analyses, demonstrating that BMI *z*-score is negatively correlated to HDL and positively and linearly correlated with TG and VLDL. BP was found to be positively related to TG. Furthermore, considering the ratios between lipids and lipoproteins, known as important predictors of the premature development of cardiovascular diseases and mortality, we studied TC/HDL, TG/HDL, LDL/HDL, and VLDL/ HDL ratios, which were higher in obese girls, compared to eutrophic controls. These results in adolescents are in line with several studies in adults, which demonstrated associations between obesity, dyslipidemia, and higher cardiovascular risks (28, 29). Nevertheless, even when it is broadly accepted that women's life events, as menopause can lead to changes in hormonal status, metabolism and lipid profile, sexual dimorphism in juvenile obesity is still poorly studied (4, 30). During puberty, the surge of gonadal hormones and of growth hormone impact all compartments of the body in terms of metabolism, and during this period, overweight individuals may present a higher risk for developing obesity-related disorders (10, 31). During the transition into puberty and then, adulthood, endocrine events are very relevant, and the differences imposed by gender may elicit heterogeneous input in the mechanisms of obesity. Yet not many studies in the literature address this aspect. Indeed, (32) point out to the fact that the vast majority of the available literature regards one gender only, and that in adolescents, there is not “obesity,” but rather “obesities,” which are gender-dependent. For that reason, the sexual dimorphism found in BP, lipids, and lipoproteins bring a new insight in childhood obesity, possibly indicating a higher risk for the development of cardiovascular diseases in female adult life.

To elucidate which mechanisms could drive hypertension and dyslipidemia in childhood obesity, we addressed serum hormone content and our results show that obesity in adolescence does not only compromise lipid and lipoprotein levels, but also hormone concentration. Our data show that hormones secreted by the endocrine pancreas, such as Insulin, C-peptide, amylin, and glucagon were increased in juvenile obesity in both sexes, compared to eutrophic counterparts. Hyperinsulinemia has been suggested to be a compensatory mechanism for an insufficient metabolic response to insulin, leading to insulin resistance (IR). Several studies in adults show that IR affects the metabolism of triglycerides and lipoproteins (33–35), thereby presenting a rationale for the correlations found in our childhood cohort, in which insulin levels were positively associated with TG and VLDL, and negatively with HDL. Also, hyperinsulinemia has been suggested to be one of the major factors causing obesity-associated hypertension (33), as increased levels of insulin can cause Sodium retention and sympathetic overactivity (13, 22). The present correlation analyses demonstrated that insulin is also positively correlated with SBP and DBP in young obese adolescents, corroborating these assumptions. Yet, insulin is not acting alone, as our data show that C-peptide released during cleavage of insulin and amylin, co-secreted with insulin, are also correlated to BP and lipid/lipoprotein alterations. Furthermore, hormones secreted by the gut enteroendocrine cells, such as GLP-1 and GIP, were increased in our juvenile obese sample. There is a well-recognized relation between microbiota dysbiosis and altered hormonal secretion (15, 36). Higher levels of these anti-inflammatory gut hormones seem to be related to short chain fatty acids (SCFAs) produced by the gut microbiota and are involved in BP regulation (37), which could explain the correlation found between GLP-1, GIP, and increased SBP values. Moreover, leptin, a hormone secreted by adipocytes was increased in obesity as compared to eutrophic adolescents, in both sexes. Interestingly, sex-dimorphism was also observed in the higher leptin content in eutrophic girls compared to eutrophic boys, as well as in obese girls compared to obese boys. This data is in line with other studies with young populations, in which adipokine levels were shown to be different according to sex-in adolescents with obesity (38, 39). Leptin influences food intake and endocrine function by acting on the hypothalamus and increasing sympathetic nervous system activation to regulate energy expenditure (13). For that reason, hyperleptinemia leading to enhanced sympathetic activation emerged as another possible factor to obesity-associated hypertension (12, 15). Our correlation data support this idea, because leptin levels were positively correlated to SBP, and as mentioned before, this was sex-dependent, as female patients showed higher values and consequently, worsened prognosis.

To better understand hormonal content alterations and the respective and multiple interactions with the sympathetic nervous system, we evaluated circulating neuropeptides in our cohort. On one hand, childhood obesity affected the expression of α-MSH, neurotensin, and melanin-concentrating hormone. In the other hand, NPY expression was sex-dependent and presented a sex-dimorphism, as obese boys had higher levels than obese girls. Neuropeptides are released in response to peripheral signals (such as hormones) to regulate appetite and energy balance (40). For example, leptin and insulin interact with hypothalamic neuropeptides NPY, MCH, and α-MSH, not only regulating appetite, but also activating the SNS, possibly contributing to obesity-related hypertension (16, 41). Nevertheless, our data only revealed a negative correlation between SBP and neurotensin, not matching previous findings (42). The low values for neurotensin in our cohort could explain the increased levels of leptin, since these neuropeptides, expressed in neuroendocrine cells of the small intestine, modulate circulating leptin content (43). In regard to NPY sexual dimorphism, lower levels of NPY found in female patients with obesity compared to male patients with obesity may be related to higher levels of leptin, inhibiting NPY production (44).

In conclusion, the significance of the study relies on the simultaneous evaluation of anthropometrical measurements, lipid and lipoproteins content, hormone levels, and neuropeptide concentration in an adolescent cohort. Our data demonstrate coexisting and inextricable linked risk factors leading to hypertension, dyslipidemia, hormonal imbalance, and neurohumoral alteration in childhood obesity. We also described a sex-dependent pattern in childhood obesity-associated dyslipidemia, which is similarly correlated with increased systolic pressure and hyperleptinemia, in female patients with obesity, solely. Considering that obesity is a preventable disease, our data on childhood obesity show several mechanisms involved in metabolic syndrome and neurohumoral alterations, bringing new possibilities for early intervention and sex-specific management to prevent cardiovascular diseases in adult life (45–48).

Nonetheless, limitations of our data should be acknowledged for future follow-up studies: the cohort was carried out with a juvenile Brazilian population sample and may be not translatable to other populations.

## Data Availability Statement

The original contributions presented in the study are included in the article/Supplementary Materials, further inquiries can be directed to the corresponding author/s.

## Ethics Statement

The studies involving human participants were reviewed and approved by the study was approved by the SCMHSP Ethics Committee (CAAE: 24552413.2.0000.5479). Written informed consent to participate in this study was provided by the participants' legal guardian/next of kin.

## Author Contributions

ES: conception and design of the study, collection of samples, molecular experiments, statistical analysis, and manuscript writing. JC-L: molecular experiments, statistical analysis, and manuscript writing. EL, TZ, DV, VO, PB, and CK: collection of samples and manuscript revision. MS: conception, design and supervision of the study, and manuscript writing. RU: conception, design and supervision of the study, and manuscript writing. All authors contributed to the article and approved the submitted version.

## Conflict of Interest

The authors declare that the research was conducted in the absence of any commercial or financial relationships that could be construed as a potential conflict of interest.
