# Supplementary material for: Sex-Dependent Dyslipidemia and Neuro-Humoral Alterations Leading to Further Cardiovascular Risk in Juvenile Obesity
Source: Front Nutr. 2021 Feb 12;7:613301. doi: 10.3389/fnut.2020.613301 (PMC7906975; doi:10.3389/fnut.2020.613301)
Supplement: Supplementary file 1 [file Data_Sheet_1.docx]

Supplementary Material

**Table S1. Quantification of serum lipid profile of the entire cohort**

|  | **Eutrophic** | | **Obese** | | **Two-way ANOVA** | | |
| --- | --- | --- | --- | --- | --- | --- | --- |
|  | **Female** | **Male** | **Female** | **Female** | **Sex** | **Obes** | **SxC** |
| **TC (mg/dL)** | 162.5 ± 6.66 | 154.9 ± 5.83 | 180.1 ± 7.21 | 162.9 ± 13.68 | 0.1236 | 0.1124 | 0.5531 |
| **TG (mg/dL)** | 69.95 ± 7.02 | 78.81 ± 8.62 | 109.5 ± 20.42 | 102.4 ± 16.1 | 0.1685 | **0.0064** | 0.5420 |
| **HDL (mg/dL)** | 56.71 ± 2.29 | 53.77 ± 3.35 | 44.6 ± 2.44 | 46.33 ± 2 | 0.9079 | **0.0008** | 0.3673 |
| **LDL (mg/dL)** | 92.1 ± 6.17 | 84.73 ± 4.78 | 118.4 ± 6.69 | 96.25 ± 12 | **0.0408** | **0.0094** | 0.2993 |
| **VLDL (mg/dL)** | 13.71 ± 1.27 | 16.36 ± 1.75 | 18.19 ± 1.56 | 20.33 ± 3.2 | 0.2573 | 0.1941 | 0.7431 |
| **Ratio TC/HDL** | 2.95 ± 0.16 | 3.06 ± 0.19 | 4.26 ± 0.27 | 3.54 ± 0.28 | 0.1777 | **0.0001** | 0.0719 |
| **Ratio TG/HDL** | 1.32 ± 0.15 | 1.61 ± 0.25 | 2.85 ± 0.73 | 2.35 ± 0.49 | 0.8634 | **0.0006** | 0.4027 |
| **Ratio LDL/HDL** | 1.69 ± 0.13 | 1.71 ± 0.15 | 2.76 ± 0.24 | 2.07 ± 0.23 | 0.0762 | **0.0003** | 0.0646 |
| **Ratio VLDL/HDL** | 0.25 ± 0.03 | 0.34 ± 0.05 | 0.43 ± 0.05 | 0.47 ± 0.09 | 0.3662 | **0.0012** | 0.4016 |

Eutrophic female, n=24; Eutrophic male, n=25; Obese female, n=22; Obese male, n=21. TC: Total Cholesterol; HDL: high-density lipoprotein cholesterol; LDL: low-density lipoprotein cholesterol; VLDL: very-low-density lipoprotein cholesterol; TG: Triglycerides; SxO: Interaction between factors (sex and obesity). Data presented as mean ± sem. Significance between the groups was tested using two-way ANOVA. Significant p-values in bold.

**Table S2. Blood pressure, lipids and lipoproteins correlations**

| **BP** | **TC** | | **TG** | | **HDL** | | **LDL** | | **VLDL** | |
| --- | --- | --- | --- | --- | --- | --- | --- | --- | --- | --- |
|  | **r** | ***p*** | **r** | ***p*** | **r** | ***p*** | **r** | ***p*** | **r** | ***p*** |
| **SBP** | 0.041 | 0.737 | **0.282** | **0.023** | -0.143 | 0.243 | 0.051 | 0.682 | 0.172 | 0.163 |
| **DBP** | 0.043 | 0.747 | 0.01 | 0.935 | 0.226 | 0.051 | -0.027 | 0.827 | -0.127 | 0.308 |

Entire cohort was considered (n=92). BP: Blood pressure; SBP: systolic blood pressure; DBP: diastolic blood pressure TC: Total Cholesterol; TG: Triglycerides; HDL: high-density lipoprotein cholesterol; LDL: low-density lipoprotein cholesterol; VLDL: very-low-density lipoprotein cholesterol. Significant differences were tested using Pearson coefficients or nonparametric Spearman correlation (r). Significant p-values (p) in bold.

**Table S3. Circulating hormones, blood pressure, lipids and lipoproteins correlations**

| **Hormones** | **SBP** | | **DBP** | | **TC** | | | **TG** | | | **HDL** | | | **LDL** | | | **VLDL** | | |  |
| --- | --- | --- | --- | --- | --- | --- | --- | --- | --- | --- | --- | --- | --- | --- | --- | --- | --- | --- | --- | --- |
|  | **r** | **p** | **r** | **p** | | **r** | **p** | | **r** | **p** | | **r** | **p** | | **r** | **p** | | **r** | **p** | |
| **Insulin** | **0.422** | **<0.0001** | **0.22** | **0.044** | | 0.043 | 0.730 | | **0.459** | **0.0001** | | **-0.396** | **0.0008** | | 0.122 | 0.324 | | **0.322** | **0.008** | |
| **Leptin** | **0.340** | **0.0015** | 0.197 | 0.073 | | 0.151 | 0.206 | | **0.254** | **0.035** | | **-0.239** | **0.043** | | 0.204 | 0.088 | | 0.214 | 0.07 | |
| **C-peptide** | **0.494** | **<0.0001** | **0.225** | **0.039** | | 0.079 | 0.508 | | **0.451** | **0.0001** | | **-0.397** | **0.0005** | | 0.187 | 0.119 | | **0.253** | **0.034** | |
| **Amylin** | **0.398** | **0.0002** | 0.151 | 0.169 | | -0.032 | 0.789 | | 0.121 | 0.323 | | -0.167 | 0.161 | | 0.012 | 0.922 | | 0.065 | 0.588 | |
| **Glucagon** | 0.117 | 0.285 | -0.011 | 0.918 | | **0.246** | **0.037** | | 0.069 | 0.571 | | -0.068 | 0.569 | | **0.269** | **0.023** | | 0.101 | 0.400 | |
| **GLP-1** | **0.415** | **<0.0001** | 0.213 | 0.052 | | 0.166 | 0.164 | | **0.293** | **0.014** | | **-0.239** | **0.043** | | 0.208 | 0.08 | | **0.258** | **0.029** | |
| **GIP** | **0.223** | **0.040** | 0.068 | 0.528 | | -0.054 | 0.651 | | 0.097 | 0.430 | | 0.026 | 0.830 | | -0.095 | 0.432 | | 0.088 | 0.465 | |
| **Ghrelin** | 0.114 | 0.297 | 0.063 | 0.571 | | 0.167 | 0.161 | | -0.072 | 0.554 | | -0.004 | 0.972 | | 0.193 | 0.108 | | -0.024 | 0.844 | |

Entire cohort was considered (n=92); GLP-1: glucagon-like peptide-1; GIP: gastric inhibitory polypeptide. Significant differences were tested using Pearson coefficients or nonparametric Spearman correlation (r). Significant p-values (p) in bold.

**Table S4. Circulating neuropeptides, blood pressure, lipids and lipoproteins correlations**

| **Neuropeptides** | **SBP** | | **DBP** | | **TC** | | | **TG** | | | **HDL** | | | **LDL** | | | **VLDL** | | |  |
| --- | --- | --- | --- | --- | --- | --- | --- | --- | --- | --- | --- | --- | --- | --- | --- | --- | --- | --- | --- | --- |
|  | **r** | **p** | **r** | **p** | | **r** | **p** | | **r** | **p** | | **r** | **p** | | **r** | **p** | | **r** | **p** | |
| **α-MSH** | -0.199 | 0.145 | -0.088 | 0.538 | | -0.097 | 0.528 | | -0.035 | 0.821 | | -0.071 | 0.639 | | -0.048 | 0.755 | | 0.046 | 0.762 | |
| **Neurotensin** | **-0.249** | **0.039** | 0.027 | 0.826 | | **-0.296** | **0.024** | | -0.227 | 0.096 | | -0.064 | 0.629 | | -0.255 | 0.056 | | -0.243 | 0.068 | |
| **NPY** | -0.068 | 0.539 | -0.009 | 0.935 | | -0.153 | 0.204 | | -0.228 | 0.062 | | 0.086 | 0.473 | | **-0.256** | **0.032** | | -0.188 | 0.119 | |
| **MCH** | -0.194 | 0.076 | **-0.299** | **0.005** | | -0.114 | 0.340 | | 0.066 | 0.587 | | 0.096 | 0.423 | | -0.142 | 0.238 | | 0.007 | 0.556 | |

Entire cohort was considered (n=92); α-MSH: α-Melanocyte-stimulating hormone; NPY: neuropeptide Y; MCH: melanin-concentrating hormone. Significant differences were tested using Pearson coefficients or nonparametric Spearman correlation (r). Significant p-values (p) in bold.
